# Literature Mining of Disease Associated Noncoding RNA in the Omics Era

**DOI:** 10.3390/molecules27154710

**Published:** 2022-07-23

**Authors:** Jian Fan

**Affiliations:** Applied Linguistics Group, School of College English Teaching and Research, Henan University, Kaifeng 475000, China; 10310045@vip.henu.edu.cn

**Keywords:** ncRNA, biomedical literature mining, deep sequencing, omics

## Abstract

Noncoding RNAs (ncRNA) are transcripts without protein-coding potential that play fundamental regulatory roles in diverse cellular processes and diseases. The application of deep sequencing experiments in ncRNA research have generated massive omics datasets, which require rapid examination, interpretation and validation based on exiting knowledge resources. Thus, text-mining methods have been increasingly adapted for automatic extraction of relations between an ncRNA and its target or a disease condition from biomedical literature. These bioinformatics tools can also assist in more complex research, such as database curation of candidate ncRNAs and hypothesis generation with respect to pathophysiological mechanisms. In this concise review, we first introduced basic concepts and workflow of literature mining systems. Then, we compared available bioinformatics tools tailored for ncRNA studies, including the tasks, applicability, and limitations. Their powerful utilities and flexibility are demonstrated by examples in a variety of diseases, such as Alzheimer’s disease, atherosclerosis and cancers. Finally, we outlined several challenges from the viewpoints of both system developers and end users. We concluded that the application of text-mining techniques will booster disease-associated ncRNA discoveries in the biomedical literature and enable integrative biology in the current omics era.

## 1. Introduction

**Figure 1:**
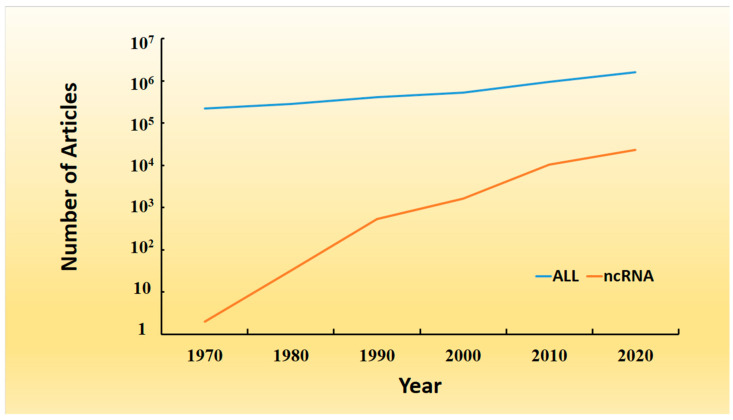
Growth of MEDLINE database. The number of articles published from the 1970s to the 2020s is shown chronologically. ‘All’ means all papers indexed in MEDLINE; ‘ncRNA’ means papers with keyword ‘ncRNA’.

High-throughput technology, such as sequencing technology, has revolutionized and accelerated biomedical research [1]. Accompanying the rapid development of scientific research, extensive literature has been published in recent decades. According to MEDLINE, a premier bibliographic database, the number of articles indexed in relation to the general or noncoding RNA (ncRNA) category significantly increased in recent decades (Figure 1). Based on this publication rate, we can estimate that there will be ~nine million articles published in the next decade. Therefore, it is difficult for researchers to comprehensively retrieve and understand all the relevant literature manually. It is also an impossible mission, even for a specialized topic, such as ncRNA.

ncRNA is a large group of RNA species that are transcribed but not coded for protein molecules [2]. Their size ranges from a dozen nucleotides, i.e., miRNA, to several thousand nucleotides, such as lncRNAs [3]. ncRNAs play fundamental regulatory roles in diverse cellular processes and diseases. The first ncRNA paper was published in English in the 1960s in English literature and reported the existence of a large RNA molecule in ribonucleoprotein [4]. Increasing research interests have since been focused on ncRNA molecules, with an increasing volume of publications. As shown Figure 1, the growth rate of articles relevant to ncRNA (searched by term ‘ncRNA’ in PubMed) is higher than that of publications in general biomedical literature, indicating that ncRNA is an active research topic. At the time of writing, there are 236,174 publications listed in PubMed when using ‘ncRNA’ as an inquiring keyword. On the other hand, sequencing technologies have generated plenty of raw datasets. A common problem is associated with systematically interpreting the sequencing results, which involve many protein-coding genes or ncRNAs. This raises the urgent need to systematically compare large data sets with all the relevant knowledge derived from published articles or raw text. Therefore, automatic text-mining techniques and systems have been developed to assist researchers in keeping up-to-date with scientific advances on a daily basis.

In this concise review, we first outlined several key concepts and methods in the text-mining field; then, we focused on systems and tools that are specifically designed for mining disease-associated ncRNA; finally, we summarized several issues that need to be addressed when making use of literature mining techniques in the omics era.

## 2. Process of Biomedical Literature Mining

We will first introduce several key vocabulary items, which are preliminaries for readers without a literature mining background.

Literature corpus: a repository of texts, either raw or tagged utilizing a set of controlled vocabularies, such as medical subject heading (MeSH) terms or Unified Medical Language System (UMLS) terms.

Terms: the named entities that are searched for. In the biomedical domain, these can refer to the names of proteins, ncRNAs, diseases, etc. Terms are usually compiled by field experts.

Concepts: a group of meaningful terms that reflect the theme of a document. Concepts can be extracted from free text or represented by a set of controlled vocabularies, such as the MeSH terms. Some authors define concepts as equivalent to “terms” and all its synonyms [5].

Natural language processing: refers to a series of approaches to analyzing text at both syntax and semantics levels. It usually involves sentence tokenization, part-of-speech tagging, word morphological analysis, etc. [6].

Machine learning: A series of linear or nonlinear statistical models to recognize the patterns or rules from a dataset. Given a corpus, linguistic processing techniques are utilized to extract qualitative and quantitative information (called ‘features’ in the text-mining field); then, these features are fed into the model for classification. Commonly used models include support vector machine (SVM) and the hidden Markov model [7,8,9].

Other key vocabularies, such as named entity recognition (NER), are introduced below in detail.

**Figure 2:**
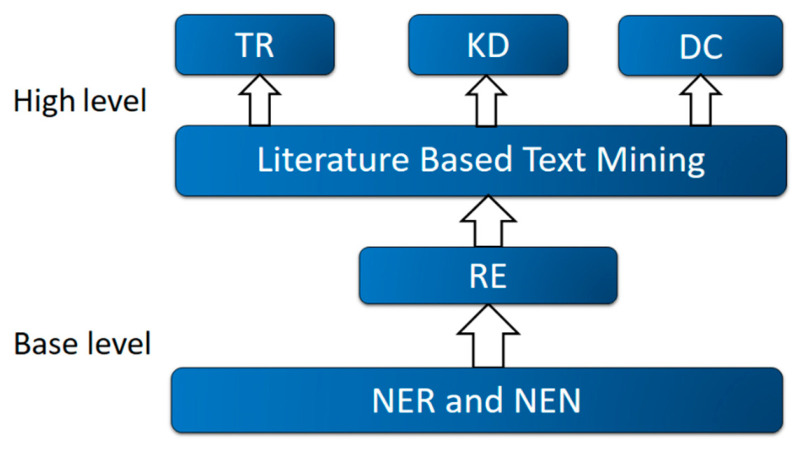
Flow chart of a typical text-mining system. Literature mining systems can be roughly divided into base level and high level. At the base level, there are three important tasks, including named entity recognition (NER), named entity normalization (NEN) and relation extraction detection (RE). Topic recognition (TR), knowledge discovery (KD) and database curation (DC) are handled at the high level.

As shown in Figure 2, the process of biomedical literature mining (BLM) can be roughly divided into base level and high level. At the base level, there are three important tasks: named entity recognition (NER), named entity normalization (NEN) and relation extraction (RE). To automatically extract information from the literature, the NER step involves identifying the relevant and professional entity (i.e., gene, ncRNA) from the sentence and phrase. There are three categories of methods associated with NER [10].

(1) Dictionary-based approaches, usually utilizing a comprehensive controlled vocabulary to directly match and identify the entity names from the documents;
(2) Semantic rule-based approaches, applying a set of handcrafted syntactic and semantic rules to fragment the sentences and capture nouns and predicates from the phrases;
(3) Statistical approaches: This family of methods first train statistical or supervised learning models, such as SVM or Markov models with labeled datasets [11,12]. Then, the pretrain models are used to predict the word and achieve the NER goal.

Each method has its advantages and disadvantages. For example, the dictionary-based method is simple and comparatively fast but needs to compile a comprehensive term set for normalization of entities, which is not trivial. Similar problems are also associated with construction of linguistic rules or patterns for identifying the named entities. Furthermore, emerging concepts cannot be effectively captured by the existing terms or semantic rules. As for machine-learning-based approaches, they are comparatively accurate but often require rich domain knowledge and are sometimes prone to producing false identification, especially when the training datasets are not representative.

**Figure 3:**
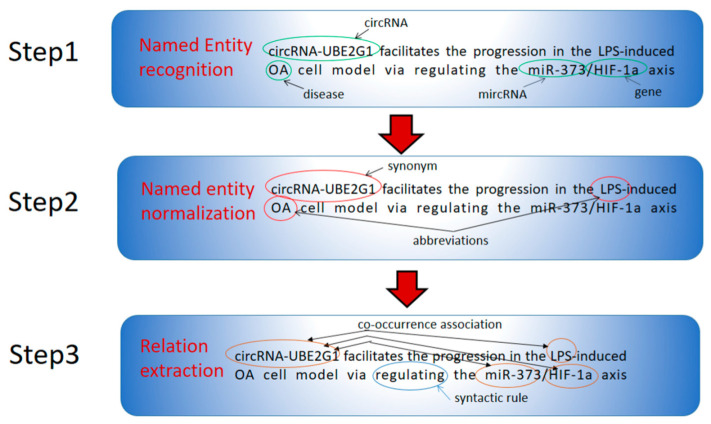
An illustration of NER, NEN and RE steps in text analysis. As an example, a real-world sentence from a circRNA research paper is used to visualize each step. In this first step, entity names, such as circRNA, gene and disease, are recognized; in step 2, word variations, such as synonyms and abbreviations, are removed by NEN methods; finally, entities’ relations are identified by co-occurrence-based, linguistic rule or machine learning methods.

In the practice of text mining, there are many linguistic variations stemming from the use of abbreviations, non-standardized names, synonyms, etc., for example, in the sentence, ‘Collectively, our data suggest that circRNA-UBE2G1 facilitates the progression in the LPS-induced OA cell model via regulating the miR 373/HIF-1a axis’ (Figure 3) [13]. circRNA-UBE2G1 is also known as ‘hsa_circ_0041557’ because it has no official name currently. Additionally, there are abbreviations in this sentence: ‘LPS’ for ‘lipopolysaccharide’ and ‘OA’ for ‘osteoarthritis’. Therefore, a matching process, known as named entity normalization, is then employed to remove these entity ambiguities. Both NER and normalization can be considered simultaneously, and they lay the foundation for many downstream analytical tasks.

A large part of biomedical research focuses on the relationship among biomolecules and their impact on diseases. It is common to see reports with the following phrases: ‘protein A bind with protein B’, ‘gene X regulates gene Y expression’ and ‘ncRNA Z play a role in disease W’; therefore, detecting and comparing the occurrences of prespecified types of relations between entity pairs is an essential task for text-mining systems. For relation extraction, there are three mainstream methods: co-occurrence-based, rule-based and machine learning. In the first category of methods, the co-occurrence of entities of interest in the same sentence or paragraph is considered to indicate a relation. As shown in Figure 3, a text miner can detect two relations: circRNA-UBE2G1/miR-373 and circRNA-UBE2G1/HIF-1a. Except for molecules’ names, co-occurrence of other types of evidence, e.g., species names, cell lines and/or experimental methods, can also be used to extract their relations. Furthermore, the times of co-existence in the same abstract or different raw texts indicate relation extraction confidence. However, significant obstacles must be overcome based on the simple co-occurrence principle. In the above example (Figure 3), a text miner can also infer an association between circRNA-UBE2G1 and LPS (lipopolysaccharide). However, circRNA-UBE2G1 and LPS have no direct relation. From this instance, it can be seen that co-occurrence-based methods, although intuitive and simple, are highly error-prone. Thus, co-occurrence of concepts in a text is often utilized as a simple baseline method, and more sophisticated approaches have been developed.

In rule-based text mining, syntactic and semantic patterns are hard-encoded in the search program. For instance, ‘ncRNA name’ <target> ‘gene or entity name’ or ‘ncRNA name’ …through <targeting> ‘gene or entity name’ are commonly used phrases in publications on ncRNA, describing that the referred ncRNA inhibits the referred gene or entity. Therefore, the specific word ‘target’ can be used to explicitly and automatically organize and link biological relations between ncRNA and genes. An example is shown in Figure 3, where a syntactic rule was used to extract the relations between circRNA-UBE2G1 and miR-373, as well as between circRNA-UBE2G1 and HIF-1a (Figure 3). Such linguistically and biological knowledge-inspired rules can be implemented into scanning programs and translated into deterministic steps to extract relations from text.

The third family of methods, machine learning based methods, view relation extraction as a classification problem. Based on annotated corpora, linguistic features around the entities in the text are collected by natural language processing techniques. This information is then fed into the classifier, e.g., SVM or naive Bayes, to extract relations. This method has been widely applied in some well-studied tasks, such as protein–protein binding extraction [14], phenotype–genotype associations [15] and drug–drug interactions [16]. Again, each method has its strengths and limitations, which are often complementary between approaches. For example, machine learning methods significantly increase accuracy often require a large amount of precompiled and annotated examples, whereas these labeled data are often expensive and require domain-specific semantic rules. Therefore, a combination of different approaches is commonly applied in modern biomedical literature mining systems.

More sophisticated and practical problems, such as topic recognition, knowledge-based discovery and database construction, are handled at the high level of BLM systems. A biological database usually collects comprehensive information on bioentities. A useful and long-lived database is expected to update its contents regularly according to the new literature. Therefore, automated text-mining tools can facilitate relevant information extraction and routine database maintenance. Relevant topic recognition can be modeled as a multilabel classification problem. Abstracts or documents are ranked by machine learning models to identify whether they are related to a selected concept, which can be represented by a MeSH term. Then, relevant texts can be further categorized based on the topic. Whereas the extraction of explicit relations and events among biomedical entities can be used to produce rich document summaries and enable answering of questions among entities, an exciting use of these methods and systems is to uncover ‘hidden’ relationships that are not present in the text but that can be inferred from existing information. This task refers to KD. Swanson’s A-B-C model forms the foundation of this field [17]. For example, it may be observed that disease A is correlated with an increase in ncRNA B. Similarly, in documents related to ncRNA B, drug C is reported to reduce the amount of ncRNA B. Therefore, although there is no direct literature about drug C’s effect on disease A, it is postulated that ncRNA B connected them. This co-occurrence-based principle is adapted naturally by many researchers to generate hypotheses, although usually performed manually. Therefore, incorporating this method explicitly in text-mining systems can accelerate this process and allow researchers to catch up with all relevant information from literature to formulate novel hypotheses. Since the early work of Swanson’s model, this association-based method has been widely used for KD, although it may identify false-positive relations. To overcome this problem, some authors developed semantic relation-based approaches involving the use of explicit linguistic rules. Because multiple semantic types exist in relation to entities or concepts, the contemporary trend is to construct a semantic graph and utilize deep learning techniques to enhance the efficiency and accuracy of KD [5].

## 3. Literature Mining of Noncoding RNA

ncRNAs pose a significant obstacle for text mining because ncRNA has unique nomenclature and terminologies. In addition, the relation among different types of ncRNAs and their interaction with protein-coding genes or diseases are complex and difficult to summarize. In addition to general-purpose systems, diverse and specific systems are needed to process the text. Below, we review selected tools useful in the different stages of text mining for disease-associated ncRNA study (Table 1). It should be noted that some of these tools combine different techniques to handle multiple tasks; therefore, we classified them according to their main applications.

### 3.1. Tools for NER, NEN and RE

The fundamental utilities of these tools are to identify the functional ncRNA species (miRNA, lncRNA, circRNA, etc.) from text and elucidate their interactions with other genes and their involvement in diseases.

miRSel [20] first compiles dictionaries for miRNA, gene and protein official names, aliases, symbols, synonyms and abbreviations. They use both general biomedical databases, such as HUGO Gene Nomenclature Committee (HGNC) (https://www.genenames.org/), gene-centered information at NCBI (Entrez Gene), Swiss-Prot Protein Database (https://www.uniprot.org/downloads) and miRNA-specific archives, such as miRBase (https://www.mirbase.org/). They have also established regular expressions to capture miRNA names in the case of miRNAs described in the literature but not yet contained in databases. The regular expressions are constructed to cover all miRNA naming conventions and frequent spelling variants. It detects sentences that contain both an miRNA and a gene and classifies them into one of the following five miRNA–gene relation categories: induction, physical target, cleavage, repression and co-expression. This classification is based on detecting the presence of specific verbs (e.g., ‘target’ and ‘repress’) that indicate these types of associations anywhere in a sentence that simultaneously mentions an miRNA and a gene. Although miRSel is an earlier attempt at text mining of miRNA, their approaches for NER and NEN are inspiring because ncRNA nomenclature is evolving, and resolving ambiguities of ncRNA names remains a key issue, especially for literature analysis of recently discovered ncRNAs types (i.e., lncRNAs and circRNA) [21].

Bagewadi [18] annotated miRNA terms and relations based on the MEDLINE database. Relation extraction between miRNAs and other identities, such as genes or diseases, can be formulated as a supervised machine learning problem. Under these circumstances, structured corpora containing true miRNA–gene relationships (which are labeled as positive samples in the text mining field) or false miRNA–gene relations (which are labeled as negative samples) are annotated manually. Language processing tools are used to generate features such as tokenization, sentence splitting, part-of-speech tagging and lemmatization from these samples. Therefore, based on several linguistic features, Bagewadi et al. proposed a linear SVM model for RE. Their annotated miRNA regular expression, corpora and text datasets publicly available, which has supported and stimulated further research on ncRNA mining.

miRTex [22]: In contrast to previous systems, miRTex uses more elaborate processing based on lexico-syntactical patterns, which perform better than the co-occurrence-based methods. miRTex has been used to extract relations from abstracts in the MEDLINE database and all the open access articles in PubMed Central. Users can query an miRNA, a gene and a disease name to search miRTex extractions via a web interface at (https://research.bioinformatics.udel.edu/miRTex/, accessed on 5 June 2022). For example, the query ‘Alzheimer’s disease’ as input returned 171 miRNA–target pairs, 315 miRNA–gene regulation pairs and 28 gene–miRNA regulation pairs. Therefore, it is a suitable tool to temporarily extract miRNA information in relation to a disease condition, especially for users without linguistics or bioinformatics expertise. However, this method is based on handcrafted rules and lists of keywords, which are not easy to generalize.

miRiaD [23]: This rule-based method assembles a set of linguistic tools to analyze the syntactic dependencies and semantic relations between miRNAs (or their features) and the related entities in the context of a disease. This method has demonstrated good performance in collecting miRNA information in the scenario of annotating miRNA–disease resources.

IBRel [24]: Although incorporating linguistic information with machine learning approaches usually outperforms co-occurrence-based methods, it requires large annotated texts to fit a classifier function [8]. However, labeled corpora are limited, especially in a still developing field, such as ncRNA. Lamurias et al. [24] adapted the concept of multi-instance learning to overcome this problem. They first used a co-occurrence approach to automatically search for an miRNA–gene pair in a sentence. However, not every co-occurrence corresponds to a true relation. Then, they grouped these pairs into bags, where at least one of the pairs is authentic but it is unknown whether all pairs in the same bag are authentic. With this method, they generated more labeled documents used for model learning. Finally, a conventional machine learning algorithm could be trained on them. IBRel has achieved better results than both co-occurrence-based approaches and an SVM model coupled with a simple linguistic kernel. Therefore, this tool is recommended when there is only a small labeled corpus available or it is difficult to compile a new training dataset.

### 3.2. Tools for Disease-Associated ncRNA Annotation

ncRNA databases contain a myriad of ncRNA information, such as sequences, interactions and expressions under disease conditions. Traditional database curation requires a domain expert to read the literature (full text or abstract) and then extract the relevant findings manually, making it both time-consuming and expensive. Given the rapid development and the explosive rate of publication of ncRNA studies, the established database should update regularly and in a timely manner. Text mining is suitable for building databases because the NER and RE techniques can automatically extract ncRNA relations with other biomolecules. Combining automatic extraction with manual annotation has become the mainstream approach to ncRNA databases, such as TarBase [25] and miRTarBase [26], using text-mining methods to provide a clue about human curation; TarBase and miRWalk [27] use co-occurrence-based methods to rank abstracts. Among them, DES-ncRNA [28] and emiRIT [29] are two recent examples.

DES-ncRNA contains relatively large vocabularies (a total of 19 topic-specific dictionaries, including antibiotics, toxins, drugs, enzymes, mutations, etc.) for extraction of associations of terms. The wide scope of this database is supports research beyond ncRNA–gene regulation. As illustrated in the original paper, DES-ncRNA not only identifies the miRNAs and lncRNAs involved in Alzheimer disease but also provides clues in relation to the potential therapeutic use of fasudil, a pharmaceutical molecule [28] However, DES-ncRNA uses only simple co-occurrences within sentences to find miRNA or lncRNA connections but lacks the robustness to capture relations beyond co-occurrence.

emiRIT, on the other hand, focuses on extracting associations between an miRNA, a gene and an extracellular location by utilizing text-mining tools that capture patterns from the syntactic structure of sentences (https://research.bioinformatics.udel.edu/emirit/, accessed on 5 June 2022). For example, the query ‘exosomes’ retrieved 192 miRNAs and 609 genes involved in 52 diseases. The linking between miRNA and cellular location is extremely useful for analysis of spatiotemporal regulation of miRNAs in the scientific literature.

We emphasize here that tools for NER, NEN and RE also useful for database annotation, as the collection of relationships between ncRNA and other biological entities, such as genes, pathways and diseases, constitute the main aspects of ncRNA database curation. For example, miRSel identifies miRNAs using a regular expression and detects miRNA–gene interactions using an RE approach; therefore, it also offers a database of literature-derived miRNAs.

### 3.3. Tools for Knowledge Discovery and Validation

Perhaps the most challenging and interesting problem is to uncover previously unknown or neglected relations with ncRNA from existing scientific literature. Then, a generated hypothesis with respect to a pathophysiological mechanism can be verified by combing omics datasets or via experiments.

LSI [30]: Although efficient in extracting direct associations, conventional co-occurrence of miRNA and disease in a sentence may overlook implicit miRNA interactions. Roy et al. developed the LSI algorithm, whereby an miRNA-by-term (keyword) frequency matrix was factorized by singular value decomposition method to extract their pairwise relationships in reduced semantic space. LSI not only accurately elucidates implicit associations between miRNAs and terms but can also group functionally similar miRNAs. LSI can be tailored by in-house corpora and newly defined terms and therefore enable users to investigate and generate new knowledge about the functional relations among ncRNAs in an interdisciplinary manner.

RWRMTN [31]: Predicting miRNAs and their regulated genes and associated diseases is an important task in the ncRNA field. The inputs of RWRMTN are miRNA–target networks and several known miRNA–disease associations; then, random walk with a restart algorithm is utilized on the network to rank candidate miRNAs. Finally, based on literature from the MEDLINE database, the author applied co-occurrence of a candidate miRNA and the disease of interest to validate their predictions. RWRMTN can visualize miRNA relations with their target genes in the interaction networks. In addition, the RWRMTN package can be downloaded for free. Thus, it is a powerful tool for users to automatically prioritize miRNA candidates according to the ranking of literature evidence, especially when interpreting omics results (See Section 3.4).

miRetrieve [32]: It is essential to gain an overview of miRNA across the affected diseases or the multiple related miRNAs in the same disease. To meet the above requirements, Friedrich et al. developed miRetrieve to identify and compare miRNAs within a selected disease or across different diseases. They first tokenized abstracts according to white space and strips punctuation. The presence of miRNA in the literature is rather complex, with variants, such as let-7; lettered suffixes, such as miR-23a/b; and miRNA enumerations, such as miR-33b/-146a/-153. miRetrieve uses regex to extract miRNA names from raw text. Then, miRNAs are associated with specific terms by frequent coexistence in the same abstracts. The authors analyzed the role of miRNAs (miR-155 and miR-21) in atherosclerosis. On the other hand, this system also efficiently elucidated the similarities and dissimilarities between atherosclerosis and lung cancer from the perspective of miRNAs. Consequently, the clinical utility, i.e., the targets of miR-155 and miR-21 in atherosclerosis as promising biomarkers, is also discussed. This example demonstrates the applicability and flexibility of miRetrieve in miRNA–disease studies.

atheMir [33]: Joppich et al. developed atheMir, a bioinformatics tool to identify miRNAs in different stages of atherosclerosis. They first scanned PubMed abstracts for co-occurrence of an miRNA and a gene based on controlled vocabularies and synonyms. They used a neural network model pretrained to find the verb connecting subject (gene or miRNA) and object (gene or miRNA). Only gene–miRNA pairs with the semantic structure in the sentence are retained. With the above elaborated steps, they filtered >99.5% of false relations. They applied the method to 28 million PubMed abstracts and found 643 miRNA–gene pairs associated with this disease. One outstanding feature of atheMir is that each abstract is grouped by five dimensions: species, disease, cell line, gene function and gene ontology; therefore, it could examine the specific miRNAs during the atherosclerotic processes and found that miRNA co-occurred in selective types of cells. Furthermore, experimentally verified miRNA–gene relations are also integrated into atheMir to facilitate generation of several interesting hypotheses. For example, miRNA-98 plays a role in monocytes, smooth muscle cells and endothelial cells. Thus, it might be responsible for macrophage attraction to endothelial cells by repressing CCL2, an immunoregulatory chemokine ligand, during inflammatory processes. atheMir provides an excellent example of mining context-based (i.e., cell-type-dependent, species- or disease-specific) miRNA–gene relations for knowledge discovery.

### 3.4. When Bibliomics Meets Multiomics

According to the PubMed, more than 1,177,000 research articles have been published in the past ten years and annotated by at least one of the ‘omics’ experiments ( using the following search terms: ‘(genomics OR proteomics OR metabolomics OR transcriptomics [MeSH])’). These omics studies generated many hypotheses, which need to be carefully examined. Starting from a classical miRNAs’ microarray experiment related to a specific disease, a list of miRNAs (usually hundreds of candidates) is differentially expressed. So, which one is the potential casual miRNA? Traditionally, an extensive literature search must be conducted and professional opinions have to be solicited from experts. However, this process has proven to be time-consuming and subjective. To accelerate this process and eliminate potential bias, a text-mining tool, such as RWRMTN, can be used to explore each candidate miRNA using information extracted from documents. This interpretation of omics results prioritizes candidates and reduces the cost of labor and time for follow-up experimental confirmation. Unfortunately, it has not been a common practice when researchers publish their results of ncRNA from high-throughput experiments. On the other hand, a hypothesis can also be generated from sequencing results first and then be validated by literature mining. For example, Henry et al. [19] analyzed the metabolites from cardiac arrest patients via ultra-high-performance liquid chromatography coupled with high-resolution tandem mass spectrometry. Lecithin cholesterol-acyltransferase (LCAT) was found to be significantly changed, which was then combined with the term ‘cardiac arrest’ as input to a text-mining system for co-occurrence association. This led to identification of LCAT as a promising drug target for cardiac arrest. Furthermore, the KD system also provided a mechanistic route of LCAT with respect to cardiac arrest, i.e., decreased LCAT activity may increase ω-3 polyunsaturated fatty acid availability in circulation, affecting cardiac arrest survival rates. We emphasize that in both scenarios, follow-up experiments are indispensable to validate the hypothesis. In addition, more research is required to investigate the best way to incorporate text-mining techniques with respect to ncRNA.

## 4. Challenges and Perspectives

After reviewing the current text-mining systems in the ncRNA field, we identified several theoretical and practical issues that should be addressed in the future. First, there is lack of regulation direction in ncRNA–gene relation extraction. For example, in the sentence showcased in Figure 3, a co-occurrence-based method cannot distinguish whether circRNA-UBE2G1 upregulates or represses miR-373/HIF-1a. Furthermore, the interaction strength between circRNA-UBE2G1 and other genes needs to be quantitatively defined and analyzed. Secondly, the task of a KD system is to exploit established scientific knowledge to generate hitherto unknown but meaningful connections. The scarcity of well-annotated ground truth datasets hinders system developers in evaluating their algorithms and results. Although several practical approaches are available, such as replication of existing medical discoveries, expert-based evaluation and comparison of results with curated databases, there is still a lack of an objective evaluation metric and unified gold-standard test datasets for KD systems [34]. Thirdly, researchers are only beginning to utilize machine-learning-based methods to identify specific terms (e.g., miRNAs) and relations; more systems are expected to be developed, as previous analysis indicates that the family of algorithms usually outperforms other methods. Lastly but not least, there are also several practical issues that need to be addressed in the future. As an increasing number of text-mining systems are deployed under circumstances such as database construction, it is preferable to develop visualization tools and user-friendly interfaces so that curators can identify relevant articles even without natural language processing expertise. In addition, text-mining systems and online web services should flexibly adapt to targeted customization in order to address specific end-user requirements [35].

## 5. Conclusions

Text mining is a new and emerging method in biomedical research and clinical practice. Expanding biomedical texts are a valuable resource for mining ncRNA–disease relations and biological insights (bibliomics). Additionally, deep-sequencing-based omics approaches have become a decisive factor in molecular medicine. The high volume of data generated from sequencing platforms also needs to be exploited to obtain meaningful results. Biomedical publications provide us with a unique dimension to interpret these omics data. With the assistance of powerful computing linguistics techniques and statistical models, bibliomics can be combined with multiple omics datasets to quickly process massive amounts of scientific information to generate and verify novel scientific hypotheses.

## Figures and Tables

**Table 1:** Selected text-mining systems and tools for ncRNA studies.

Tools	Tasks				Methods					PMID
	NER & NEN	RE	DC	KD	Dictionary-Based	Co-Occurrence	Semantic Approaches	Rule-Based	Machine Learning	
Bagewadi et al. [18]	Y	Y				Y	Y		Y	26535109
miRSel	Y	Y			Y	Y				20233441
miRTex	Y	Y			Y		Y			26407127
miRiaD	Y	Y					Y			27216254
IBRel	Y	Y							Y	28263989
DES-ncRNA			Y	Y		Y				28387604
emiRIT			Y	Y				Y		34048547
miRetrieve				Y		Y	Y			34988440
LSI				Y		Y				27766940
RWRMTN				Y		Y				32539680
atheMir				Y		Y		Y	Y	31378854
Henry et al. [19].				Y		Y				34250435

Note: Main tasks of literature mining systems include named entity recognition (NER), named entity normalization (NEN) relation extraction (RE), database curation (DC) and knowledge discovery (KD).

## Data Availability

Not applicable.
